# Combined Effect of Phage phT4A and Pressure-Based Strategies in the Inhibition of *Escherichia coli*

**DOI:** 10.3390/antibiotics11020211

**Published:** 2022-02-07

**Authors:** Carla Pereira, João F. Marques, Sílvia Reis, Pedro Costa, Ana P. Martins, Carlos A. Pinto, Jorge A. Saraiva, Adelaide Almeida

**Affiliations:** 1 Department of Biology and CESAM, University of Aveiro, 3810-193 Aveiro, Portugal; csgp@ua.pt (C.P.); pedrommrscosta@ua.pt (P.C.); 2 Department of Chemistry and LAQV-REQUIMTE, University of Aveiro, 3810-193 Aveiro, Portugal; jf.marques@ua.pt (J.F.M.); silviareis@ua.pt (S.R.); ana.patricia.martins@ua.pt (A.P.M.); carlospinto@ua.pt (C.A.P.)

**Keywords:** phages, hyperbaric storage, refrigeration, combined therapies, bacterial regrowth

## Abstract

The major concern regarding the bacteriophage (or phage) therapy approach is the regrowth of bacteria after treatment, a consequence of the emergence of phage-resistant mutants. However, this limitation can be overcome by combining different therapies. In this study, the potential of combining phage phT4A with pressure storage (HS) to enhance the control of *Escherichia coli* and bacterial regrowth after treatment was evaluated. For that, the combining effect of phage phT4A and HS was studied and compared with storage at atmospheric pressure (AP) under refrigeration (4 °C, RF) and room temperature (RT). Initially, the effect of high hydrostatic pressure (200, 300 and 400 MPa) and HS (75 MPa), as well as refrigeration in phage phT4A viability, was determined. However, a considerable phage inactivation was verified at 200 MPa and so only HS at 75 MPa was further studied for combined treatment. The combined treatment with phage phT4A and HS was more efficient (reduction of 2.5 log CFU/mL after 7 days of storage) than phage phT4A (*E. coli* concentration was similar to that of the bacterial control after 7 days of storage) and HS (reduction of 1.8 log CFU/mL after 7 days of storage) applied individually. The combination of phage phT4A with refrigerated storage did not decrease *E. coli* levels. However, both the combination of phage with HS and the treatment with HS at 75 MPa effectively reduced *E. coli* concentration and prevented its regrowth. Phage phT4A viability was slightly affected during HS; however, the efficiency of the combined treatment phage-HS was not compromised. Further studies are needed to validate these findings in food products.

## 1. Introduction

Foodborne pathogens are a serious worldwide public health problem despite all advances made in food sanitation techniques and pathogen surveillance [1,2]. Pathogenic *E. coli* is one of the main bacterial contaminants associated with foodborne infections [3,4]. It spreads almost exclusively through fecal contamination of food and water, but can also be transmitted through cross-contamination or human contact during food processing [1]. The main route of exposure appears to be the consumption of contaminated food, notably raw or undercooked minced meat, raw milk and fresh produce [1]. *E. coli* is a non-pathogenic commensal bacterium known for its versatility and variety, due to its ability to colonize human and non-human gastrointestinal systems [5]. However, some *E. coli*, namely, *E. coli* O157:H7, produce toxins that cause diseases with severe symptoms [6]. *E. coli* O157:H7 can produce Shiga or Shiga-like toxins that can cause haemolytic uremic syndrome and hemorrhagic colitis in humans [6,7]. Shiga toxins are responsible for several diseases such as bloody diarrhoea and can lead to kidney failure due to severe haemolytic-uremic syndrome [6,7].

Methods such as thermal pasteurization, high-pressure processing, refrigeration, and chemical additives can improve food safety by reducing microbial populations in food to different degrees [8]. However, these approaches can affect the nutritional value and deleterious impact on organoleptic qualities of foods [8]. Despite advances made in improving foods safety, including antimicrobial interventions and HACCP systems, *E. coli* O157:H7 contamination continues to be a significant problem and its incidence has been increasing in recent years [9], which highlights the need for further research and development of novel antimicrobial treatments and interventions. A promising alternative for the prevention and elimination of bacterial pathogens is the use of phages as antibacterial agents. 

Phages, viruses that only infect bacteria, are natural bactericides, are self-replicating, have high specificity targeting to their bacterial host and do not modify the normal properties of food [10]. In 2006, the Food and Drug Administration (FDA) from the USA approved ListShield™, a phage-based product to control *Listeria* in meat and poultry products [11]. Since then, the regulator has significantly increased the research and development of new phage-based technologies for the control of different pathogens in post-harvest foods [11]. Resulting in the development and approval of phage products such as EcoShield™, SalmoLyse^®^, and ShigaShield™ from Intralytix Inc. (Baltimore, MD, USA); SalmoPro^®^ from PhageLux (Montreal, QC, Canada) and PhageGuard S™, PhageGuard Listex™and PhageGuard E™ from Micreos Food Safety (Wageningen, The Netherlands) for biotherapeutic application in food [8,12]. The application of lytic phages has been suggested as a method of mitigating *E. coli* in foods such as beef [13,14,15], tomato [15], spinach [15], broccoli [15], cantaloupes [16], lettuce [16,17,18], bivalves [19,20], milk [21,22], lamb and mutton [23]. Nonetheless, the major concern of this alternative therapy is the growth of phage-resistant mutants [24]. Their development has been attributed to genetic alterations [25]; however, some studies indicate that it is due to phenotypic resistance that these bacterial populations manage to remain viable in the presence of phages, as they remain genetically sensitive to them [26]. 

Pressure as a measure for food preservation is being increasingly used in the food industry for nonthermal pasteurization by high-pressure processing (HPP) that makes use of elevated pressures (commercially up to 600 MPa). Nevertheless, HPP is still a more technological approach and combining it with other technologies for the inactivation of microorganisms is worth studying [27,28]. In addition, hyperbaric storage (HS) at room temperature (RT) has been proposed as a potential alternative to refrigeration for food preservation. HS is an innovative preservation method capable of preserving food products under pressure (between 25 and 220 MPa) at RT, for days, weeks, or months [29,30,31]. This preservation method has potential because of the efficiency with which it preserves food and the potential energy savings it could achieve. Some studies demonstrated that HS/RT can preserve fruit juices [32,33], raw bovine meat [34], and convenience foods [35] more efficiently than conventional refrigeration. However, hyperbaric storage does not always guarantee total microorganism inactivation and it is in our interest to improve inactivation by combining other methodologies [30,31,36]. The combination of phage application with other nonthermal processing techniques, such as hyperbaric storage, could be used to prevent bacterial regrowth after treatment, but the interaction of these treatments is unknown.

Thus, this work evaluated the combined effect of phage therapy (using phage phT4A) and HS (75 MPa) on *E. coli* inactivation, its effect on bacterial regrowth and how it compares with storage at atmospheric pressure (AP) under refrigeration (4 °C) and RT. The effect of HS at 75 MPa, high hydrostatic pressure (200, 300 and 400 MPa) as well as refrigeration on phT4A phage viability was also determined.

## 2. Materials and Methods

**Figure 1:**
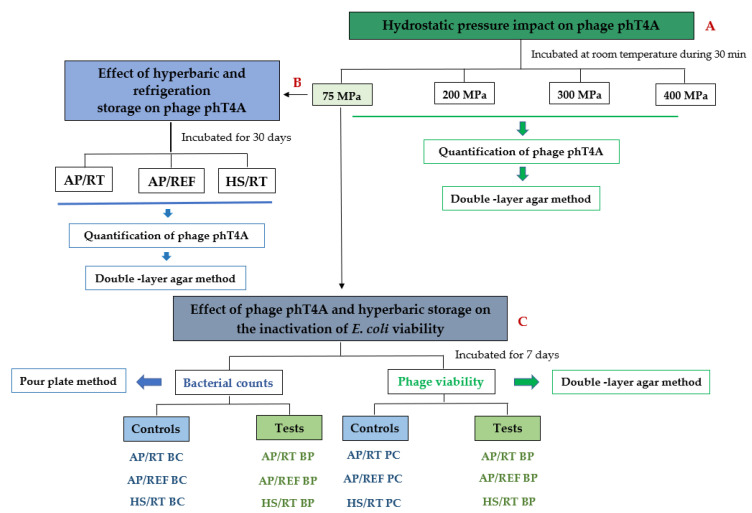
Schematic diagram of the experimental design. (**A**) Effect of hydrostatic pressure (75, 200, 300 and 400 MPa) and processing time on phage phT4A viability (see Section 2.3); (**B**) Phage phT4A viability under atmospheric pressure at room temperature (AP/RT) and with refrigeration (AP/REF), and under 75 MPa pressure at RT (HS/RT) for 30 days (see Section 2.4); (**C**) Effect of phage phT4A and HS on *E. coli* inactivation and phage phT4A viability after 7 days of storage at different conditions: under atmospheric pressure at room temperature (AP/RT) and 4 °C (AP/REF), and under a 75 MPa pressure at room temperature (HS/RT) (see Section 2.5). BC, bacteria control; PC, phage control; BP, bacteria plus phage.

Initially, the effect of high hydrostatic pressure (200, 300 and 400 MPa) and HS (75 MPa) on phage phT4A viability was assessed for 30 min (Figure 1, Section 2.3). Next, the effect of HS as well as refrigeration in phage phT4A viability was monitored for 30 days (Figure 1, Section 2.4). Then, the combined effect of phage phT4A and HS (75 MPa) on *E. coli* inactivation was evaluated and a comparison with storage at atmospheric pressure (AP) under refrigeration (4 °C) and RT was made (Figure 1, Section 2.5).

### 2.1. Bacterial Strains and Growth Conditions

The bacterial strain *Escherichia coli* (ATCC 13706) was used in this study. The bacterium was grown in Tryptic Soy Broth (TSB; Liofilchem, Roseto degli Abruzzi, Italy). The fresh plate bacterial culture was maintained in Tryptic Soy Agar medium (TSA; Liofilchem, Roseto degli Abruzzi, Italy) at 4 °C. Before each assay, one isolated colony was aseptically transferred to 10 mL of Tryptic Soy Broth and was grown overnight at 37 °C at 120 rpm stirring. An aliquot of this culture (100 µL) was aseptically transferred to 10 mL of fresh TSB and grown overnight at 37 °C to reach an optical density (O.D. 600 nm) of 0.8 (HaloDB-20; DynamicaScientific, Livingston, UK), corresponding approximately to 10^9^ cells per mL.

### 2.2. Preparation of Phage phT4A and Enrichment

Phage phT4A was isolated from the sewage network of Aveiro (station EEIS9 of SIMRIA Multi Sanitation System of Ria de Aveiro) in a previous study [37]. It was identified as a double-stranded DNA phage belonging to the family *Myoviridae* (accession number KX130727) [37]. 

The phage suspension was prepared using *E. coli* (ATCC 13706) as the host from a previously prepared phage stock in SM buffer (0.1 M NaCl (Sigma, St. Louis, MO, USA), 8 mM MgSO_4_ (Sigma, St. Louis, MO, USA), 20 mM Tris-HCl (Sigma, St. Louis, MO, USA), 2% (*w*/*v*) gelatin, pH 7.5). Five hundred microliters of the phage stock were added to 30 mL TSB and 1 mL of the bacterial host in the exponential growth phase. The suspension was grown overnight and incubated at 37 °C at 80 rpm. The lysate was centrifuged at 13,000 rpm for 10 min at 4 °C. To remove intact bacteria or bacterial debris, the supernatant was filtered through a polyethersulphate membrane with a 0.22 µm pore size (Merck-Millipore, Darmstadt, Germany). The phage suspension was stored at 4 °C until use and the titer was determined by the double-layer agar method [38].

Successive dilutions of the phage suspension were performed in phosphate-buffered saline (PBS) (137 mmol^−1^ NaCl (Sigma; St. Louis, MO, USA), 8.1 mmol^−1^ Na_2_HPO_4_·2H_2_O (Sigma; St. Louis, MO, USA), 2.7 mmol^−1^ KCl (Sigma; St. Louis, MO, USA) and 1.76 mmol^−1^ KH_2_PO_4_ (Sigma; St. Louis, MO, USA), pH 7.4) and 500 μL of each dilution were added to 100 μL of fresh *E. coli* culture, mixed with 5 mL of TSB 0.6% top agar layer (30 g/L TSB (Liofilchem, Roseto degli Abruzzi, Italy), 6 g/L agar (Liofilchem, Roseto degli Abruzzi, Italy), 0.05 g/L CaCl_2_ (Sigma, St. Louis, MO, USA), 0.12 g/L MgSO_4_ (Sigma, St. Louis, MO, USA), pH 7.4) and poured over a TSA plate. The plates were incubated at 37 °C for 8–12 h. After incubation, the number of plaques was counted and the results were expressed as plaque-forming units per milliliter (PFU)/mL. 

### 2.3. Hydrostatic Pressure Impact on Phage phT4A Viability

Phage suspensions were transferred to 0.4 mL polyethylene tubes. Three independent samples were analyzed, each with three sub-samples (*n* = 9) and two replicates per sub-sample (*n* = 18). The tubes containing the sub-samples were placed in low permeability polyamide/polyethylene bags (PA/PE-90, Ideiapack, Comércio de Embalagens Lda, Viseu, Portugal) with 70% ethanol, thermo-sealed and pressurized. The conditions tested were 75 MPa/30 min, 200 MPa/30 min, 300 MPa/30 min and 400 MPa/30 min in a hydrostatic press (high-pressure system U33, Institute of High-Pressure Physics, Warsaw, Poland) at RT and the pressurization fluid was a mixture (60:40) of water and propylene glycol (DOWCAL™, Dow). Non-pressurized controls were also included in the different experiments.

The pressurized and unpressurized samples were tittered by the double-layer agar method [38]. Successive dilutions of the phage suspension were performed in PBS and 100 µL of each dilution were added to 100 µL of fresh *E. coli* culture, mixed with 5 mL of TSB 0.6% top agar layer and placed over a Petri dish containing solid TSA. The plates were incubated for 12 h at 37 °C. The survivor number was reported as log PFU/mL and the values were used to determine the high-pressure reduction effectiveness (RE), following the equation RE = log (N_0_/N), where N_0_ and N represent the number of viable cells in the unpressurized and pressurized suspensions, respectively. The detection limit of this method was 10 PFU/mL.

### 2.4. Effect of Hyperbaric and Refrigeration Storage on Phage phT4A Viability

Phage suspensions were transferred to 0.4 mL polyethylene tubes as described in Section 2.3. Three independent samples were analyzed, each with three sub-samples (*n* = 9) and two replicates per sub-sample (*n* = 18). 

Phage reduction was determined using single phage suspensions of phage phT4A (10^8^ PFU/mL) at room temperature (AP/RT), under HS at 75 MPa at room temperature (HS/RT) and under refrigeration (4 °C) (AP/REF) for up to 30 days. The HS experiments for the 75 MPa at naturally varying room temperatures (~21 °C) were carried out in high pressure (FPG13900 Stansted Fluid Power Ltd., SFP, Harlow, UK) in pressure vessels with 35 mm inner diameter for 520 mm high (0.4 L volume).

Aliquots of test samples were collected at time 0 and after 1, 10 and 30 days of storage. Phage concentration was determined in duplicate through the double-agar layer method [38] after an incubation period of 6–8 h at 37 °C and was expressed as plaque-forming units per milliliter (PFU/mL). 

### 2.5. Hyperbaric Storage Experiments

*E. coli* reduction (10^7^ CFU/mL) was determined using single phage suspensions of phage phT4A (10^7^ PFU/mL) (AP/RT BP), under HS at 75 MPa (HS/RT BP) at room temperature and under refrigeration (4 °C) (AP/REF BP) for up to 7 days. During the experiment, four control samples at atmospheric pressure (0.1 MPa) were included: (i) *E. coli* at the same temperature conditions as HS (AP/RT BC); (ii) phage phT4A at the same temperature condition as for HS (AP/RT PC); (iii) *E. coli* under refrigeration (4 °C) (AP/REF BC) and (iv) phage phT4A under refrigeration (4 °C) (AP/REF PC). Two more control samples were also included under HS at 75 MPa at room temperature: (i) an *E. coli* control (HS/RT BC) and (ii) a phage phT4A control (HS/RT PC). 

Bacterial suspensions were transferred to 0.4 mL polyethylene tubes. Three independent samples were analyzed, each with three sub-samples (*n* = 9) and two replicates per sub-sample (*n* = 18). The tubes containing the sub-samples were placed in low permeability polyamide/polyethylene bags (PA/PE-90, Ideiapack, Comércio de Embalagens Lda, Viseu, Portugal) with 70% ethanol and then thermo-sealed for the hyperbaric storage experiments. 

HS experiments for the 75 MPa at naturally varying room temperatures (~21 °C) were carried out in high pressure (FPG13900 Stansted Fluid Power Ltd., SFP, Harlow, UK) in pressure vessels with 35 mm inner diameter for 520 mm high (0.4 L volume).

Control and test samples were incubated in the same conditions. Aliquots of test samples and controls were collected at time 0 and after 0.5, 1.5, 2.5, 4.5 and 7 days of storage. Phage concentration was determined in duplicate through the double-agar layer method [38] after an incubation period of 6–8 h at 37 °C and was expressed as plaque-forming units per milliliter (PFU/mL). Bacterial concentration was determined in duplicate in TSA medium after 24 h at 37 °C and was expressed as colony-forming units per millilitre (CFU/mL).

### 2.6. Statistical Analysis

The statistical analysis of data was performed using GraphPad Prism 7.04 software (San Diego, CA, USA). Normal distribution of the data was checked by the Kolmogorov–Smirnov test and the homoscedasticity was assessed by Levene’s test. Significance was accepted at *p* < 0.05. Tukey’s multiple comparison test was used for pairwise comparison of the means. The significance of bacterial and viral concentrations during treatments along the experiments was tested using two-way analysis of variance (ANOVA) and Bonferroni post hoc tests. The significance of the differences recorded for bacterial and phage concentration was evaluated by comparing the results of test samples of each experimental treatment with the correspondent control for the different sampling times. 

## 3. Results

### 3.1. Hydrostatic Pressure Impact on Phage phT4A Viability

**Figure 2:**
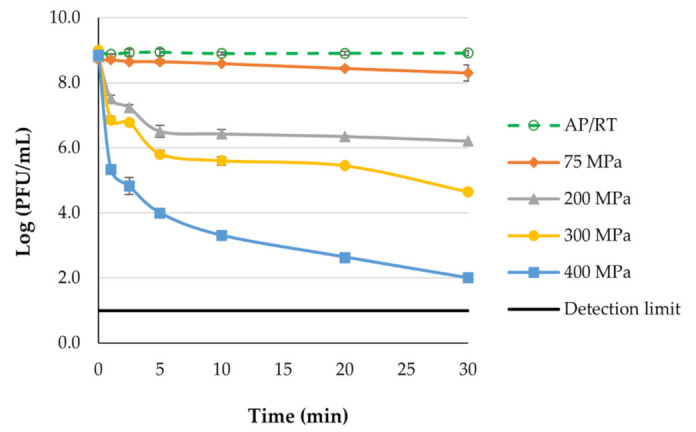
Effect of hydrostatic pressure (75, 200, 300 and 400 MPa) on phage phT4A viability. AP/RT, atmospheric pressure room temperature.

Phage density in the non-pressurized suspensions (NTS) remained constant during the 30 min of the experiment (ANOVA, *p* > 0.05; Figure 2).

The increase in pressure from 75 to 400 MPa significantly improved the inactivation factor during treatment (ANOVA, *p* < 0.05). The maximum phage phT4A reduction was 0.63, 2.73, 4.23 and 6.89 log PFU/mL for suspensions treated at 75, 200, 300/30 min, respectively (Figure 2 and Table 1). At 400 MPa, phage phT4A was inactivated to below the detection limit. The inactivation in samples pressurized at 200, 300 and 400 MPa was significantly different (ANOVA, *p* < 0.05) from non-pressurized samples during the different processing times (Figure 2 and Table 1). The increase in processing time significantly improved (ANOVA, *p* < 0.05) inactivation in the samples pressurized at 300 and 400 MPa. However, phage density under a 75 MPa pressure was similar (ANOVA, *p* > 0.05) to the non-pressurized samples (Figure 2 and Table 1). Since at 200 MPa pressure a considerable phage inactivation already occurred, only the study of hyperbaric storage at 75 MPa was carried out.

### 3.2. Effect of Hyperbaric and Refrigeration Storage on Phage phT4A Viability

**Figure 3:**
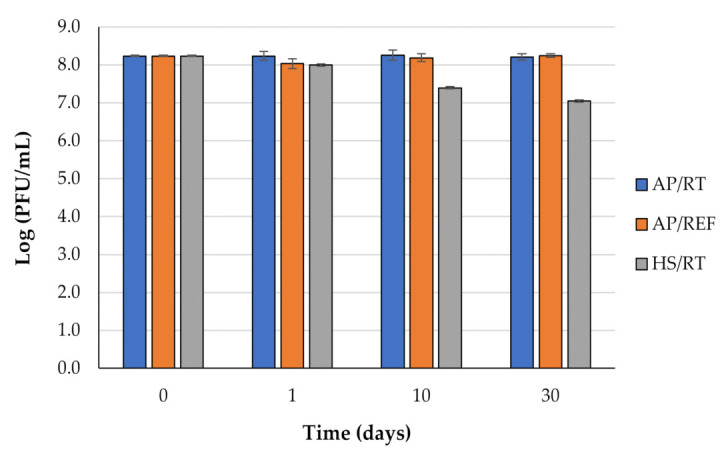
Phage phT4A viability under atmospheric pressure at room temperature (AP/RT) and refrigeration (AP/REF), and 75 MPa pressure at RT (HS/RT) for 30 days.

In the control stored at ~21 °C (AP/RT), phage phT4A concentration remained constant (ANOVA, *p* > 0.05) for 30 days (Figure 3). Refrigeration at 4 °C (AP/REF) maintained phage viability similar to the initial value (ANOVA, *p* > 0.05) for 30 days (Figure 3). HS at 75 MPa (HS/RT) caused a slight viral reduction of about 0.84 and 1.19 log PFU/mL (ANOVA, *p* < 0.05) after 10 and 30 days, respectively (Figure 3).

### 3.3. Effect of Phage phT4A and Hyperbaric Storage on the Inactivation of E. coli

**Figure 4:**
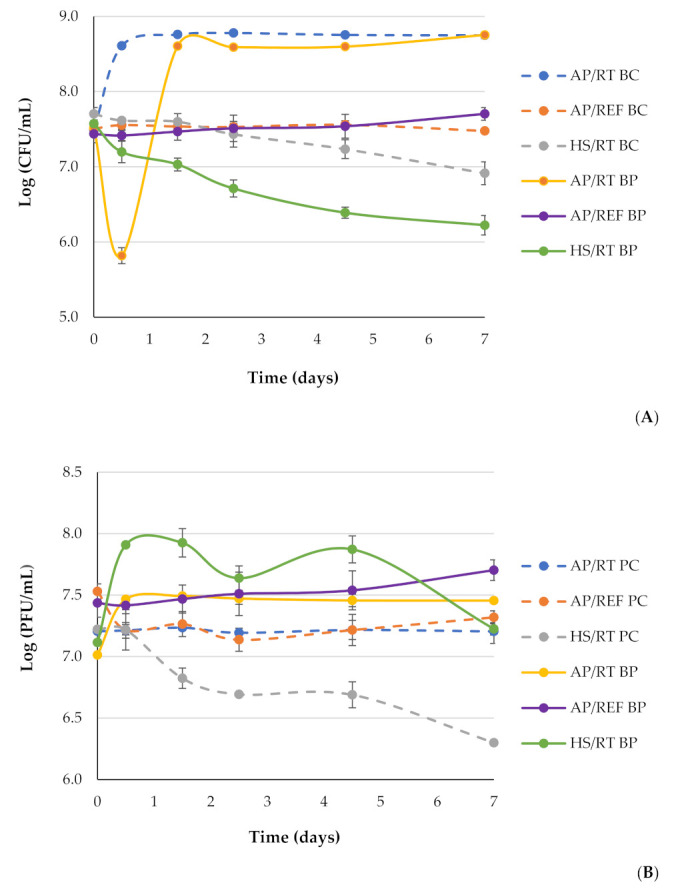
Bacterial counts of *E. coli* with phage phT4A (**A**) and phage phT4A viability (**B**) during the 7 days of storage at different conditions: under atmospheric pressure at room temperature (AP/RT) and 4 °C (AP/REF) and 75 MPa pressure at (HS/RT). (**A**) Bacterial concentration: BC, bacteria control; BP, bacteria plus phage. (**B**) Phage concentration: PC, phage control; BP, bacteria plus phage. Values represent the mean of three experiments; error bars represent the standard deviation. Dashed lines: controls and solid lines: test samples.

After 7 days of storage at atmospheric pressure and room temperature (AP/RT BC), *E. coli* density increased by 1.23 log CFU/mL (ANOVA, *p* < 0.05) (Figure 4A). The maximum *E. coli* reduction with phage phT4A (AP/RT BP) was 2.79 log CFU/mL, achieved after 0.5 days of storage (Figure 4A). However, *E. coli* regrowth was observed after 1.5 days, reaching bacterial densities similar (ANOVA, *p* > 0.05) to those obtained in the bacterial control stored at atmospheric pressure and room temperature (AP/RT BC). *E. coli* reduction after the 7 days was statistically similar (ANOVA, *p* > 0.05), when compared to bacterial control stored at atmospheric pressure and room temperature (AP/RT BC) (Figure 4A).

Refrigeration at 4 °C maintained the *E. coli* counts similar to the initial value (ANOVA, *p* > 0.05) during the 7 days of the experiment (AP/REF BC) (Figure 4A). When the phage was incubated in the presence of *E. coli* under refrigeration (AP/REF BP), the bacterial count also remained similar to the initial value and similar to the treatment with the phage alone (AP/REF BC) (Figure 4A).

In the combined treatment (HS/RT BP), the bacterial density was significantly lower (ANOVA, *p* > 0.05) than that obtained in the treatments with HS at 75 MPa (HS/RT BC) and room temperature (AP/RT BC) (Figure 4A). HS alone (HS/ RT BC) enabled a maximum *E. coli* reduction of 1.8 log CFU/mL achieved after 7-day storage at 75 MPa, when compared to bacterial control stored at atmospheric pressure and room temperature (AP/RT BC). However, after 1.5- and 2.5-day storage at 75 MPa, the reduction level was already high (1.16 and 1.35 log CFU/mL; ANOVA, *p* < 0.05, respectively) (Figure 4A). When the phage was combined with HS at 75 MPa (HS/RT BP), the maximum level of *E. coli* inactivation was 2.50 log CFU/mL (ANOVA, *p* < 0.05) achieved after 7 days of storage. During the experiment, significant differences (ANOVA, *p* > 0.05) were observed between the combined treatment with phage phT4A and HS at 75 MPa (HS/RT BP) and the treatment with only HS at 75 MPa (HS/RT BC). In the combined treatment (HS/RT BP), as well as in the treatment with HS at 75 MPa (HS/RT BC), no regrowth of bacteria was observed during the experiment (Figure 4A).

At atmospheric pressure, phage density remained constant throughout the experiment at both room temperature (AP/RT PC) and 4 °C (AP/REF PC) (ANOVA, *p* > 0.05). When phage phT4A was incubated in the presence of *E. coli* at room temperature (AP/RT BP), an increase of 0.49 log PFU/mL was observed in the phage concentration (Figure 4B). However, when phage phT4A was incubated in the presence of *E. coli* at 4 °C (AP/REF BP), phage concentration remained constant (ANOVA, *p* > 0.05) for the 7 days of the experiment (Figure 4B). 

Phage density after HS at 75 MPa (HS/RT PC) decreased 0.92 log PFU/mL after 7 days of storage (Figure 4B). When the phage was combined with HS at 75 MPa (HS/RT BP), phage density slightly increased (0.45 log PFU/mL) after 0.5 days of storage, then remained constant between 1.5 and 4.5 days. After 4.5 days, phage phT4A density decreased by 0.54 PFU/mL (Figure 4B).

## 4. Discussion

Phage therapy is an eco-friendly alternative approach to preventing and controlling pathogenic bacteria in the food industry. However, despite several studies attesting for its efficacy, the development of bacterial regrowth is still a general concern [5,37,39,40,41]. Some studies have shown that combining therapies can increase treatment effectiveness and prevent bacterial regrowth [5,41,42]. However, the interaction between phages and pressure, whether high-pressure processing or hyperbaric storage (75 MPa), is unknown, particularly regarding the prevention of bacterial regrowth, a focus of this study.

The application of high-pressure processing (HPP) treatment reduced phage phT4A concentration in magnitudes dependent on pressure and treatment time (Figure 2). As pressure (75 to 400 MPa) and treatment time increased, so did the effective reduction in phage phT4A. Phage concentration decreased 0.63, 2.73, 4.23 and 8.89 log PFU/mL when treated at 75, 200, 300 and 400 MPa/30 min, respectively. The mechanism of virus inactivation by hydrostatic pressure is not yet well understood. However, it has been shown that hydrostatic pressure treatments do not affect the viral nucleic acids [43,44,45]. Viral inactivation comes from the inability of viral particles to bind to the host cell due to the denaturation of the capsid proteins essential for this attachment [44,45]. Additionally, viral inactivation depends on process- (pressure, temperature and time) and product- (food matrix composition, pH, salt and water activity) related parameters [46,47]. Generally, the inactivation of microorganisms increases logarithmically with increasing pressure and the tail effect occurs with increasing treatment times [48,49]. Thus, since HPP caused considerable phage inactivation already at 200 MPa, only hyperbaric storage at 75 MPa was further studied. 

Phage phT4A significantly decreased (2.79 log CFU/mL) *E. coli* concentration after 0.5-day storage relative to the bacterial control (without phage addition) stored at atmospheric pressure and room temperature (AP/RT BC). Some studies have successfully prevented or controlled *E. coli* with phages [16,22,37,50,51]. However, according to the results of this study, the use of phage phT4A did not prevent bacterial regrowth after treatment. When phage phT4A was used alone, bacterial regrowth was observed after 0.5 days of storage, reaching bacterial densities such as those obtained in the bacterial control stored at atmospheric pressure and room temperature (AP/RT BC) after 1.5 days of storage. Similar results have been obtained by other authors [5,37,41]. Lopes et al. (2018) showed that phage ELY-1 was effective against *E. coli* during the first 12 h of treatment [5]. However, an *E. coli* regrowth was observed, reaching similar values to those obtained in the bacterial control after 36 h of incubation [5]. In another study, when phage ECA2 was used, *E. coli* regrowth was observed after 4 h of incubation [37]. In the future, further studies will be needed to evaluate the frequency of emergence of spontaneous phage-resistant *E. coli* and to identify the bacterial receptors used by phage phT4A. There are several mechanisms involved in resistance, namely (i) alteration or loss of bacterial cell surface receptors; (ii) receptor blockage by the bacterial extracellular matrix; (iii) inhibition of phage DNA penetration; (iv) production of modified restriction endonucleases that effectively hydrolyze phage DNA; (v) inhibition of intracellular phage assembly; or (vi) CRISPR-Cas’s systems aided by retrons (bacterial defense systems against phage infection complemented with retrons molecules, hybrid DNA structures composed of half RNA and half ssDNA, acting as guards that ensure bacterial survival upon phage infection) [52]. However, the mutations affecting phage receptors on the bacterial cell surface represent the most frequent cause of resistance [52].

HS at 75 MPa (HS/RT BC) showed higher *E. coli* inactivation than conventional refrigeration (AP/REF BC), achieving viable cell reductions of 1.8 log CFU/mL, while in conventional refrigeration, *E. coli* concentration remained constant. In fact, several studies proved that HS-RT preserved fruit juices [33], meat [34,53,54], fish [55], milk products [56,57] and convenient foods [31,35] more efficiently than conventional refrigeration. In recent years, HS has been looked at as a possible method for food preservation by inhibiting microbial growth, while maintaining the overall physicochemical parameters of food [34,56,57]. However, while only minor microbial inactivation was achieved with this HS, the combination of natural antimicrobial agents (such as phages) with HS can improve decontamination efficiency and enhance food products safety and quality. The use of phages is not allowed in Europe. However, the U.S. Food and Drug Administration (FDA) has approved two phage preparations, designated “EcoShield™” developed by Intralytix, Inc. (Baltimore, MD, USA) and PhageGuard E from Micreos Food Safety (Wageningen, The Netherlands) to reduce *E. coli* O157:H7 in various foods [12]. Moreover, in developed countries such as the USA, Canada, Australia and New Zealand, food law policies have already been updated regarding phage applications [58]. 

For the combination of therapies to be successful, each agent must have different bacterial targets, such as the case of phages and hyperbaric storage, contributing to an increased inactivation. In this study, the bacterial density reduction achieved by the combined action of phage phT4A and hyperbaric storage (2.5 log CFU/mL after 7 days of storage) was higher than the phage (*E. coli* concentration was similar to that of bacterial control after 7 days of storage) and hyperbaric storage (1.8 log CFU/mL after 7 days of storage) applied individually. In the combined treatment (HS/RT BP) and the treatment with HS at 75 MPa (HS/RT BC), no bacterial regrowth was observed during the experiments. When phage phT4A was used alone, after 0.5 days of storage, *E. coli* regrowth was observed, reaching bacterial densities similar to those obtained in the bacterial control stored at atmospheric pressure and room temperature (AP/RT BC). The combination of phage phT4A with refrigeration storage (AP/RT BC) does not increase the efficacy of bacterial inactivation. In the combined treatment (AP/REF BP), *E. coli* concentration was similar to that obtained in conventional refrigeration (AP/REF BC).

The efficiency of the combined therapy depends on the ability of the host bacteria to replicate the phages, which is affected by low temperature. In fact, this combined treatment (AP/REF BP) affects phage phT4A production. When the phage was combined with refrigeration, the number of phages in the presence of the host remained constant (phage concentration similar to that of phage control). Temperature is thus crucial for lytic phage viability [59,60,61,62,63], affecting phage attachment, genome injection and multiplication [64]. Only a few viral particles can inject their genetic material into bacterial host cells at low temperatures and therefore only these few are involved in the amplification phase. At 4 °C, bacterial enzymatic activity is very low and this is also why phages do not burst cells at that temperature. When phage phT4A was incubated in the presence of *E. coli* at room temperature (AP/RT BP), an increase of 0.49 log PFU/mL was observed in the phage concentration. In the combined treatment of phage phT4A with HS (HS/RT BP), phage density slightly increased (0.45 log PFU/mL) after 0.5 days of storage, then remained constant between 1.5 and 4.5 days. After 4.5 days, phage phT4A density slightly decreased (Figure 4B), but the number of phages remaining was enough for bacterial inactivation.

In the future, it will be essential to understand the efficacy of the combined treatment of phages with HS in different food matrices. One of the challenges while using phages as biocontrol agents in different food products is the effect of the food matrix on phage stability and efficacy [65]. Some studies have reported greater reductions in bacterial loads in liquid foods than in solid or semi-solid food matrices [66,67]; this is because the liquid matrix allows greater phages diffusion and facilitates access to the bacterial target. One solution to compensate for this is to apply high initial phage concentrations to the solid food product [68]. However, even high phage concentrations may not be sufficient for prolonged protection during storage because different factors, such as food proprieties (pH, storage temperature), may affect phage survival and lytic properties [69,70]. As such, further studies using different pressures, phage and bacteria concentrations, different phages and bacteria, and even phage cocktails, are necessary to validate these results. It should be highlighted that only one strain of *E. coli* was studied and the current results need to be further validated for other strains, including different strains of *E. coli* O157:H7.

## 5. Conclusions

High-pressure processing showed to be a reliable methodology to inactivate the phage phT4A, with the inactivation levels augmenting with the increase of the pressure level and holding time.

The combined treatment of phage phT4A with HS effectively reduced *E. coli* concentration and prevented bacterial regrowth. Although phage phT4A viability was affected by HS, the efficiency of the phage-HS combination was not compromised. The results of this study emphasize the importance of testing the efficacy of new approaches to inactivate bacteria in the food industry.

## Figures and Tables

**Table 1:** Reduction in phage phT4A after different hydrostatic pressure conditions.

Samples Conditions	Log PFU/mL	Reduction Effectiveness (Log PFU/mL)
AP/RT/5 min	8.94 ± 0.11	-
AP/RT/20 min	8.90 ± 0.05	-
AP/RT/30 min	8.91 ± 0.08	-
75 MPa/5 min	8.65 ± 0.06	0.29 ^a,d,e,f^
75 MPa/20 min	8.44 ± 0.05	0.46 ^a,d,e,f^
75 MPa/30 min	8.30 ± 0.24	0.61 ^a,d,e,f^
200 MPa/5 min	6.50 ± 0.18	2.44 ^b,c,e,f^
200 MPa/20 min	6.34 ± 0.03	2.56 ^b,c,e,f^
200 MPa/30 min	6.20 ± 0.02	2.71 ^b,c,e,f^
300 MPa/5 min	5.81 ± 0.04	3.13 ^b,c,d,f^
300 MPa/20 min	5.45 ± 0.03	3.45 ^b,c,d,f^
300 MPa/30 min	4.65 ± 0.08	4.26 ^b,c,d,f^
400 MPa/5 min	4.00 ± 0.12	4.94 ^b,c,d,e^
400 MPa/20 min	2.64 ± 0.08	6.26 ^b,c,d,e^
400 MPa/30 min	ND	7.91 ^b,c,d,e^

AP/RT, atmospheric pressure at room temperature. ND, Not Detected (below the limit of detection, 1 log PFU/mL). ^a^ Not significantly different (*p* > 0.05) from the phage phT4A non-pressurized. ^b^ Significantly different (*p* < 0.05) from the phage phT4A non-pressurized. ^c^ Significantly different (*p* < 0.05) from the phage phT4A pressurized at 75 MPa. ^d^ Significantly different (*p* < 0.05) from the phage phT4A pressurized at 200 MPa. ^e^ Significantly different (*p* < 0.05) from the phage phT4A pressurized at 300 MPa. ^f^ Significantly different (*p* < 0.05) from the phage phT4A pressurized at 400 MPa.

## Data Availability

Not applicable.
